# Mesh smoothing algorithm based on exterior angles split

**DOI:** 10.1371/journal.pone.0232854

**Published:** 2020-05-12

**Authors:** Yongqing Hai, Siyuan Cheng, Yufei Guo, Shaojing Li

**Affiliations:** 1 Department of Mechanics and Engineering Science, College of Engineering, Peking University, Beijing, China; 2 State Key Laboratory of Hydraulic Engineering Simulation and Safety, Tianjin, China; University of New South Wales, AUSTRALIA

## Abstract

Since meshes of poor quality give rise to low accuracy in finite element analysis and kinds of inconveniences in many other applications, mesh smoothing is widely used as an essential technique for the improvement of mesh quality. With respect to this issue, the main contribution of this paper is that a novel mesh smoothing method based on an exterior-angle-split process is proposed. The proposed method contains three main stages: the first stage is independent element geometric transformation performed by exterior-angle-split operations, treating elements unconnected; the second stage is to offset scaling and displacement induced by element transformation; the third stage is to determine the final positions of nodes with a weighted strategy. Theoretical proof describes the regularity of this method and many numerical experiments illustrate its convergence. Not only is this method applicable for triangular mesh, but also can be naturally extended to arbitrary polygonal surface mesh. Quality improvements of demonstrations on triangular and quadrilateral meshes show the effectiveness of this method.

## 1. Introduction

Nowadays, mesh casts an irreplaceable role in extensive fields as an effective way of discretization. In advanced manufacturing field, there is finite element analysis for structures, fluids, electromagnetics and thermodynamics; in biological field, there is medical imaging, organs 3D printing and surgical simulation; in the field of computer vision and graphics, there is Simultaneous Localization and Mapping (SLAM), graphical semantic segmentation, Geographic Information System (GIS), video special effects and so on. There are many mature mesh generation approaches, such as marching cubes [1], Delaunay [2], advancing front [3, 4], frame field [5] and so on. But the mesh quality cannot meet all the requirements among these applications, and the quality influence final performances to a great extent. For instance, meshes with poor quality can lead to precision decline in finite element analysis, distortions in geometry surface modeling and bad effects even failure in graphics rendering. Such situations put forwards higher requirements for mesh quality. For this purpose, mesh quality improvement needs to be introduced and the research about this technique has also attracted a lot of attention. This paper presents a mesh smoothing method based on an exterior-angle-split (EAS) process to improve mesh quality.

This paper is organized as follows. Section 2 reviews related previous work. Section 3 illustrates the rule of element transformation for triangular mesh and the global triangular mesh smoothing strategy. And properties of this algorithm are also discussed in this section. Section 4 applies this smoothing method to quadrilateral mesh and arbitrary polygon mesh. Results and discussions of some numerical tests are presented in Section 5. Section 6 presents the conclusion and future work of this paper.

## 2. Related work

Methods to improve mesh quality can be roughly divided into geometric methods, topological methods and combined methods. **Geometric methods** focus on nodes’ geometric positions without changing connectivity of nodes. Among geometric methods, there are also two subclasses, optimization-based methods and smoothing methods. Relying on mathematical models, optimization-based methods [6–9] regard mesh quality as a form of energy. The quality can be characterized as an energy formula relying on mathematical models, so that the issue of quality improvement is converted into an optimization problem. Through minimizing the energy function, these methods can gain better global quality but usually a time-consuming work. While smoothing methods make a balance between mesh quality and computation efficiency, which is also what this paper concentrates on. Such easy-implement methods can meet the quality requirement of applications in most cases. Elements surrounding a node can be seen as a *ring*. An idea of smoothing is to relocate the central point based on its *ring*. The well-known Laplacian smoothing [10, 11] moves the central point to the average of its surrounding vertices’ positions. On the basis of this there are also modifications [12–14] to avoid generating negative elements, known as constrained Laplacian smoothing. The outer outline of a *ring* is a polygon. Based on the polygon of a node’s *ring*, angle-based smoothing [15] takes an iterative scheme to move the central point to the average of intersection points of adjacent interior angle bisectors of the polygon. It can obtain better angle distribution and maximize the minimum angle. Meanwhile, GETMe [16, 17] starts another viewpoint of mesh smoothing based on element transformation. This viewpoint focuses on each element shape instead of the central point position of its *ring*. SSO [18] is also an element transformation method, which optimizes mesh quality with the assistance of the height of element.

**Topological methods** improve mesh quality through the ways of changing the connectivity of nodes as well as the number of nodes. Basic operations including edge split, edge collapse and edge swap can be usually seen in [19, 20]. Small polyhedral reconnection [21] is proposed to eliminate poorly-shaped element, which is also can be used in surface mesh.

**Combined methods** have both geometric operations and topological operations. Centroidal Voronoi Tessellation is the concept of generating points at mass centroids of the corresponding Voronoi tessellations derived from a triangulation, which is applied to mesh optimization in [22–24]. Optimal Delaunay Triangulations [25, 26] improve quality through minimizing a defined interpolation error.

This paper presents a new geometric method based on exterior angle split to improve mesh quality. The proposed method achieves quality improvement mainly relying on a linear element transformation and an assembly strategy.

## 3. Smoothing algorithm for triangular mesh

This section presents an exterior-angle-split algorithm for triangular mesh smoothing. Element geometric transformation is to make each element equilateral. And the final result is obtained with a weighted assembly strategy. The regularity for element transformations can be proved on the theory level and numerical experiments illustrate its convergence after finite times of iterations. As for triangular mesh, this paper selects the ratio of two times incircle radius (2r) and circumcircle radius (R) of the triangle as quality criteria, denoted as γ.

### 3.1 Transformation of single element

EAS method regards elements unconnected in the first two stages, therefore, each element transformation is independent to each other. A transformation for one element does not influence its adjacent elements at all, this will be specified in Section 3.3.

A local Cartesian right-handed coordinate system is built to describe the transformation where z-axis coincides with outer normal vector of the element. Nodes of an element are recorded in a counterclockwise direction, denoted as *p*_*m*_, *m*ϵ{0,1,2}. A counterclockwise directed edge is denoted as ***p***_***mod*(*m*−1,3)**_***p***_***m***_, and a clockwise directed edge can be denoted as ***p***_***mod*(*m*+1,3)**_***p***_***m***_. An interior angle can be denoted as *α*_*m*_, *m*ϵ{0,1,2}, whose subscript is the same as the subscript of the corresponding node *p*_*m*_. And the superscript stands for the current stage. For example, $p_{m}^{\left(0\right)}$ is the original position and $p_{m}^{\left(1\right)}$ is the position after the first stage.

**Fig 1:**
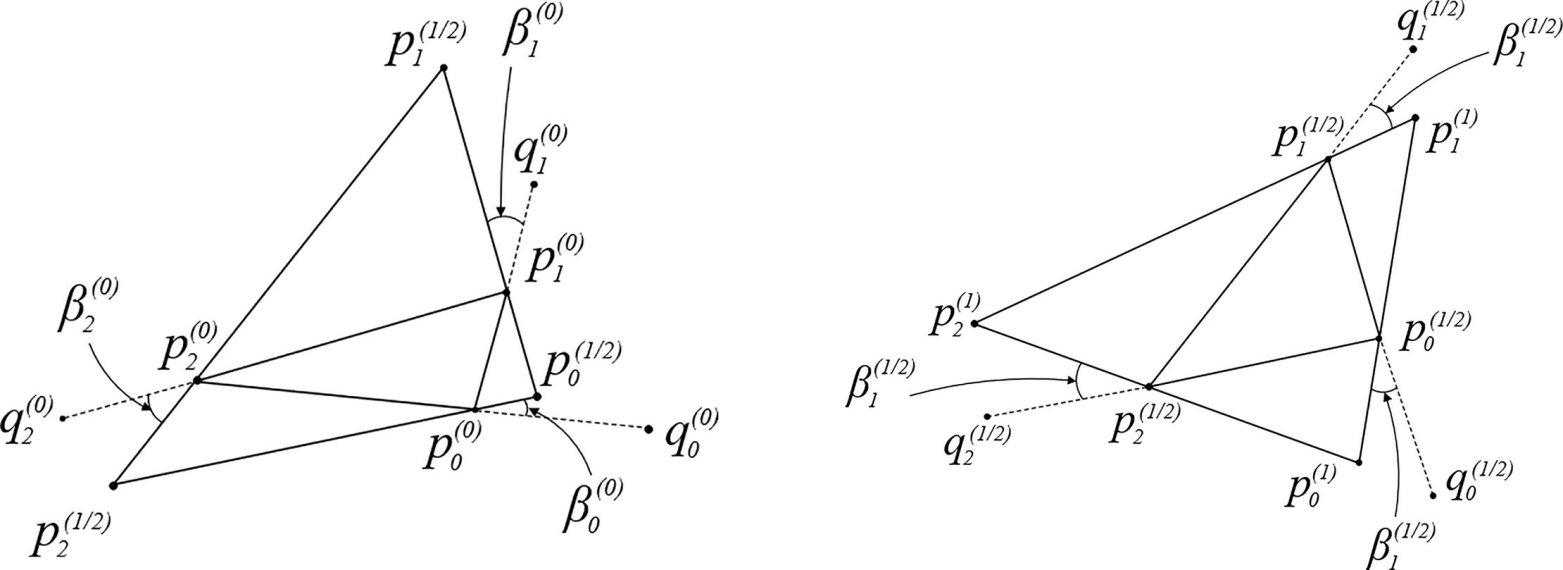
Substages of the first stage for EAS method. a) the first substage in counterclockwise direction; b) the second substage in clockwise direction.

The first stage EAS method consists of two substages, a counterclockwise transformation substage and a clockwise transformation substage. Take the counterclockwise substage as the first substage for instance as depicted in Fig 1A. First, exterior angles can be constructed by the extension lines of counterclockwise directed edges $p_{mod(m-1,3)}^{\left(0\right)}p_{m}^{\left(0\right)}$. For a clearer description, we define a virtual point $q_{m}^{\left(0\right)}$ at the infinite position of extension line, hence the extension line can be denoted as $p_{m}^{\left(0\right)}q_{m}^{\left(0\right)}$. Next, a parameter *t*, 0<*t*<1, is introduced to define a split line which splits each exterior angle into two angles. One of the angles denoted as $\beta_{m}^{\left(0\right)}$ is expressed as $t∙∠q_{m}^{\left(0\right)}p_{m}^{\left(0\right)}p_{mod(m+1,3)}^{\left(0\right)}$. According to geometric relations, $\beta_{m}^{\left(0\right)}$ can also be expressed as $t∙(∠\alpha_{mod(m-1,3)}^{\left(0\right)}+∠\alpha_{mod(m+1,3)}^{\left(0\right)})$. Then, three intersection points of split lines can be calculated with arithmetic. Finally, endpoints $p_{m}^{\left(0\right)}$ of the directed edges are moved to their corresponding intersection points $p_{m}^{\left(1/2\right)}$. The second substage is a reperform of the first substage but in clockwise direction as depicted in Fig 1B, where $p_{m}^{\left(1\right)}$ can be obtained.

**Fig 2:**
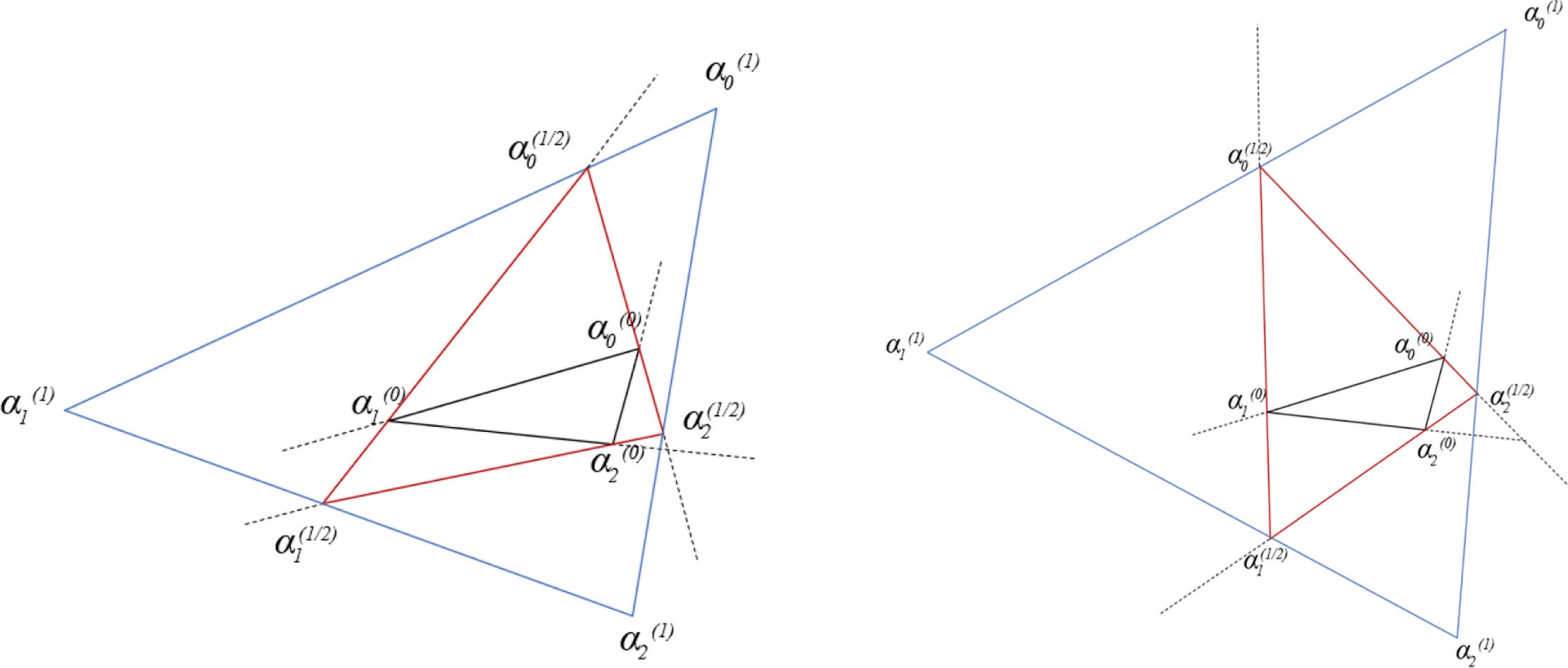
Operations of the first stage of EAS method for triangular mesh. a) shows EAS transformation when t is 0.25; b) shows EAS transformation when t is 0.5. Black dotted lines are extended line of directed edge; triangles of black solid line are original elements, triangles of red solid line are elements after the first substage and triangles of blue solid line are elements after the second substage.

The successive two substages are in the opposite direction to each other to rotation offset. It doesn't matter that which direction is the first, and it is just customary to take the counterclockwise as the first one. Fig 2 shows transformations with *t* taking the value of 0.25, 0.5 respectively, where $\alpha_{m}^{\left(0\right)}$ is the original interior angle, $\alpha_{m}^{\left(1/2\right)}$ is the interior angle after the first substage and $\alpha_{m}^{\left(1\right)}$ is the interior angle after two substages.

$$p_{m}^{\left(2\right)}={bc}^{\left(0\right)}+\mu (p_{m}^{\left(1\right)}-{bc}^{\left(1\right)}),\;m=0,1,2$$ (1)

**Fig 3:**
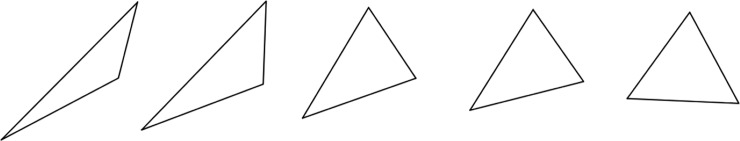
The geometric transformation process of a triangle element.

Although an element can obtain a better shape after EAS transformation as shown in Fig 2, necessary shrinking operation which is the second stage of EAS method have to be taken to offset the scaling and displacement without shape change. The second stage selects either the ratio between the perimeter or the ratio between the square root of area before and after transformation as shrinking coefficient *μ*. Eq 1 specifies the shrinking operation with shape preserving and barycenter preserving, where *bc*^(0)^, *bc*^(1)^ are the barycenter before and after the transformation of the first stage, $p_{m}^{\left(1\right)},\;p_{m}^{\left(2\right)}$ are nodes’ positions before and after shrinking. The second stage is to preserve the positive influence generated by the first stage and get rid of the negative influence. It drags the element back to its original barycenter and maintains its original size, meanwhile, a better shape is preserved. Fig 3 shows the shape-changing process of a triangular element of poor quality.

### 3.2 Regularity

Through observing changes of interior angles, the underlying laws can be discovered. We use the symbols the same as Fig 1 and Fig 2 to clarify the property of EAS transformation. The interior angle after the first stage of EAS method is denoted as $\alpha_{m}^{\left(1\right)}$. Since the second stage of EAS method does not change the shape as well as the interior angle, the value of $\alpha_{m}^{\left(1\right)}$ is preserved after the second stage. As illustrated in Fig 1A, $∠p_{mod(m+1,3)}^{\left(0\right)}p_{m}^{\left(0\right)}p_{m}^{\left(1/2\right)},\;∠p_{m}^{\left(1/2\right)}p_{mod(m+1,3)}^{\left(0\right)}p_{m}^{\left(0\right)}$ and $∠p_{m}^{\left(0\right)}p_{m}^{\left(1/2\right)}p_{mod(m+1,3)}^{\left(0\right)}$ are three interior angles of $△p_{mod(m+1,3)}^{\left(0\right)}p_{m}^{\left(0\right)}p_{m}^{\left(1/2\right)}$, and $∠p_{m}^{\left(0\right)}p_{m}^{\left(1/2\right)}p_{mod(m+1,3)}^{\left(0\right)}$ is $\alpha_{m}^{\left(\frac{1}{2}\right)}$.

$$\alpha_{m}^{\left(\frac{1}{2}\right)}=\pi -(1-t)∙{(\alpha }_{mod(m+1,3)}^{\left(0\right)}+\alpha_{mod(m-1,3)}^{\left(0\right)})-t{∙(\alpha }_{m}^{\left(0\right)}+\alpha_{mod(m-1,3)}^{\left(0\right)})$$ (2)

$$\left\{\alpha^{\left(\frac{1}{2}\right)}\}=K_{+}\{\alpha^{\left(0\right)}\}=\left[\begin{array}{ccc} 1-t & t & 0\\ 0 & 1-t & t\\ t & 0 & 1-t \end{array}\right]\{\alpha^{\left(0\right)}\right\}$$ (3)

$$\left\{\alpha^{\left(1\right)}\}=K_{-}\{\alpha^{\left(\frac{1}{2}\right)}\}=\left[\begin{array}{ccc} 1-t & 0 & t\\ t & 1-t & 0\\ 0 & t & 1-t \end{array}\right]\{\alpha^{\left(\frac{1}{2}\right)}\right\}$$ (4)

According to the theorem that an exterior angle is equal to the sum of two nonadjacent interior angles in a triangle, $∠p_{m}^{\left(1/2\right)}p_{m}^{\left(0\right)}p_{mod(m+1,3)}^{\left(0\right)}$ is equal to $\left(1-t)∙{(\alpha }_{mod(m+1,3)}^{\left(0\right)}+\alpha_{mod(m-1,3)}^{\left(0\right)}\right)$, and $∠p_{m}^{\left(1/2\right)}p_{mod(m+1,3)}^{\left(0\right)}p_{m}^{\left(0\right)}$ has the value of $t{∙(\alpha }_{m}^{\left(0\right)}+\alpha_{mod(m-1,3)}^{\left(0\right)})$. Hence, the new interior angle $\alpha_{m}^{\left(\frac{1}{2}\right)}$ can be calculated naturally by Eq 2. Eq 3 describes this substage in the form of matrix.

Similarly, the second substage illustrated in Fig 1B can be described as Eq 4, where $\alpha_{m}^{\left(1\right)}$ is calculated. The two successive substages of EAS element transformation can be represented as Eq 5, where K is the transit matrix, *K* = *K*_+_∙*K*_−_.

$$\left\{\alpha^{\left(1\right)}\}=K\{\alpha^{\left(0\right)}\}=\left[\begin{array}{ccc} {2t}^{2}-2t+1 & -t^{2}+t & -t^{2}+t\\ -t^{2}+t & {2t}^{2}-2t+1 & -t^{2}+t\\ -t^{2}+t & -t^{2}+t & {2t}^{2}-2t+1 \end{array}\right]\{\alpha^{\left(0\right)}\right\}$$ (5)

Clearly, the element in row i column j is equal to element in row j column i, making K a symmetric matrix. According to the theorem of spectral decomposition, K has three eigenvalues and three orthogonal eigenvectors. And its eigenvalues *λ*_*m*_, *m*ϵ{0,1,2}, are 3*t*^2^−3*t*+1, 3*t*^2^−3*t*+1,1, respectively, which means K is a positive definite matrix. After performing the normalization of eigenvectors, $\eta_{m}=\rho_{m}/\sqrt{\rho_{m}∙\rho_{m}}$, the orthogonal matrix *Φ* = [*η*_0_ *η*_1_ *η*_2_] consisting of normalized eigenvectors is obtained. The spectral decomposition of symmetric Matrix K can be expressed as Eq 6, where *I*_*n*_ denotes a 3×3 matrix with the only non-zero coefficient in row n column n, and (*I*_*n*_)_*n*,*n*_ = 1.

$$K=\Phi \Lambda \Phi^{H}=\sum_{n=0}^{2}\lambda_{n}(\Phi I_{n}\Phi^{H})$$ (6)

A series of transformation can be naturally expressed as {*α*}^(*l*)^ = *K*^*l*^{*α*}^(0)^, where *l* is the number of element transformations. On condition that if 0<*t*<1, then 0<*λ*_0_ = *λ*_1_<1, we can get lim_*l*→∞_*λ*_0_^*l*^ = *λ*_1_^*l*^ = 0. This leads to that *λ*_2_ = 1 is dominant item for EAS transformation as shown in Eq 7.

$$K^{l}=\sum_{n=0}^{2}{\lambda_{n}}^{l}(\Phi I_{n}\Phi^{H})=\sum_{n=2}^{2}{\lambda_{n}}^{l}(\Phi I_{n}\Phi^{H})=\lambda_{2}∙\Phi I_{2}\Phi^{H}=\frac{1}{3}\left[\begin{array}{ccc} 1 & 1 & 1\\ 1 & 1 & 1\\ 1 & 1 & 1 \end{array}\right]$$ (7)

As is known to all, the sum of interior angles of a triangle is 180°. Eq 7 implies that a series of EAS transformation can average interior angles of an element to 60° i.e. each element tends to be a regular triangle.

### 3.3 Assembly

In the first two stages, each element is treated unconnected, and transformations and shrinking operations are conducted independently for each isolated element, therefore, one element’s operations will not deteriorate others’ quality. By the end of the second stage, an integral mesh may be transformed into a set of isolated elements. Section 3.3 elaborates the assembly strategy for isolated elements to gain the final result which is also the third stage of EAS method.

**Fig 4:**
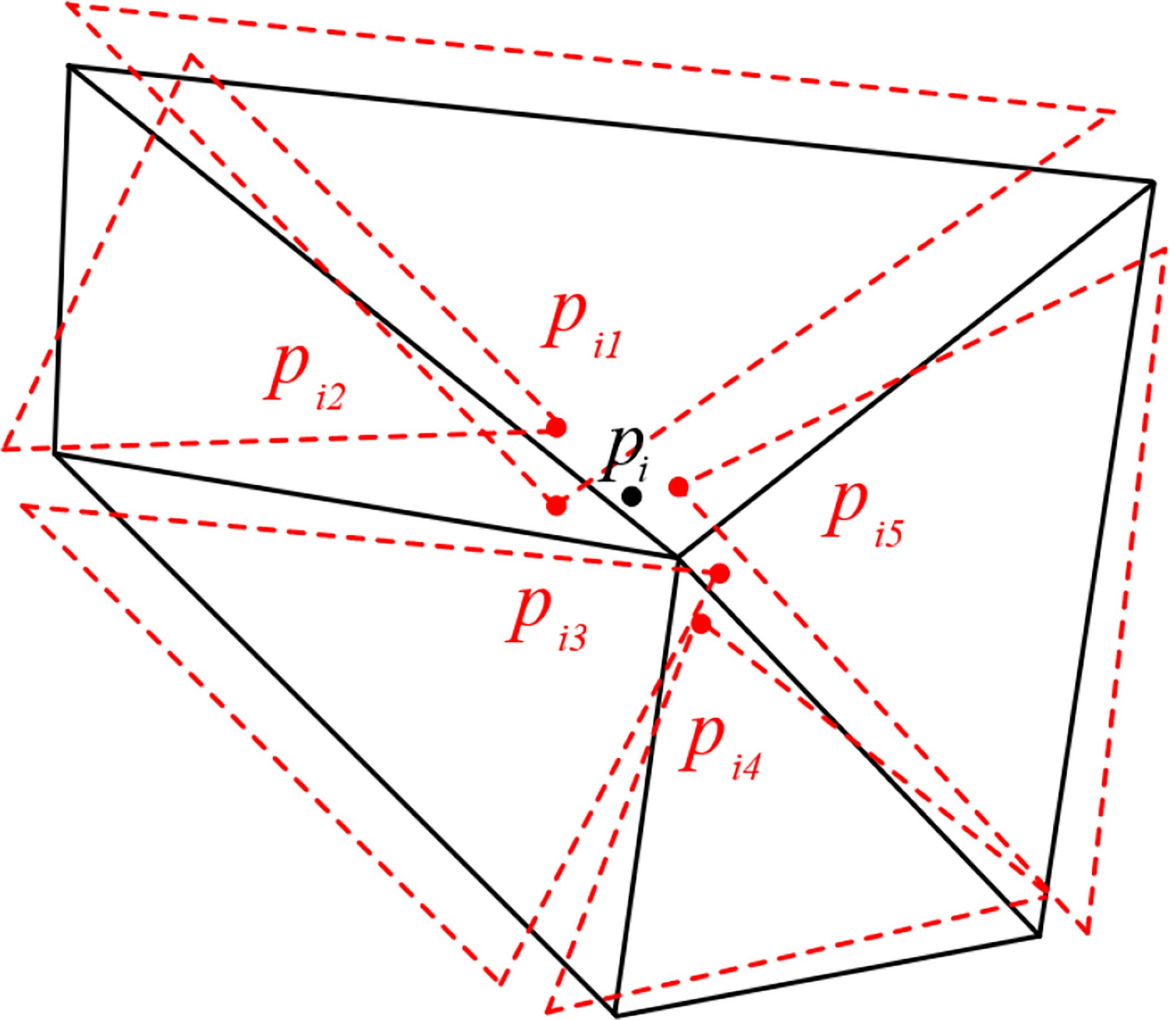
Assembly operation of one node in EAS smoothing. The node *p*_*i*_ has the valence of 5, its 5 original included elements are represented in black solid lines; after the first two stages of EAS method, 5 elements are transformed into 5 isolated ones in red dotted lines, and 5 replicas of *p*_*i*_ are represented in red thick point, denoted as *p*_*ij*_.

A node with the valence of *v* on the original mesh means that it is included by its *v* adjacent elements. Naturally, after two stages of EAS method, *v* replicas of this original node exist its *v* unconnected included elements as illustrated in Fig 4. In order to determine the final position of nodes, a weighted strategy is selected to relocate the position of each node based on the positions of its replicas as well as the sizes and quality changes of its included elements, as represented in Eq 8.

$$p_{i}^{\left(3\right)}=\frac{1}{2}∙\sum \left(\frac{\Delta \gamma_{ij}}{\sum \Delta \gamma_{ij}}∙p_{ij}\right)+\frac{1}{2}∙\sum \left((1-\frac{s_{ij}}{\sum s_{ij}})∙p_{ij}\right)$$ (8)

In Eq 8, $p_{i}^{\left(3\right)}$ is the final position of node with a global index i, *p*_*ij*_ is the position of its corresponding replica with local index j, *s*_*ij*_ is the area of original element, and Δ*γ*_*ij*_ is the quality change compared to the original element with local index j. Through the third stage, isolated elements are assembled into an integral one again. Clearly, the time cost of the whole EAS method is linear, whose time complexity is *O*(*n*), where the *n* is the number of elements.

EAS method is a mutually independent work for each element, which provides access to parallel programming for simultaneous transformations and assembly on the global mesh.

### 3.4 Boundary treatments

**Fig 5:**
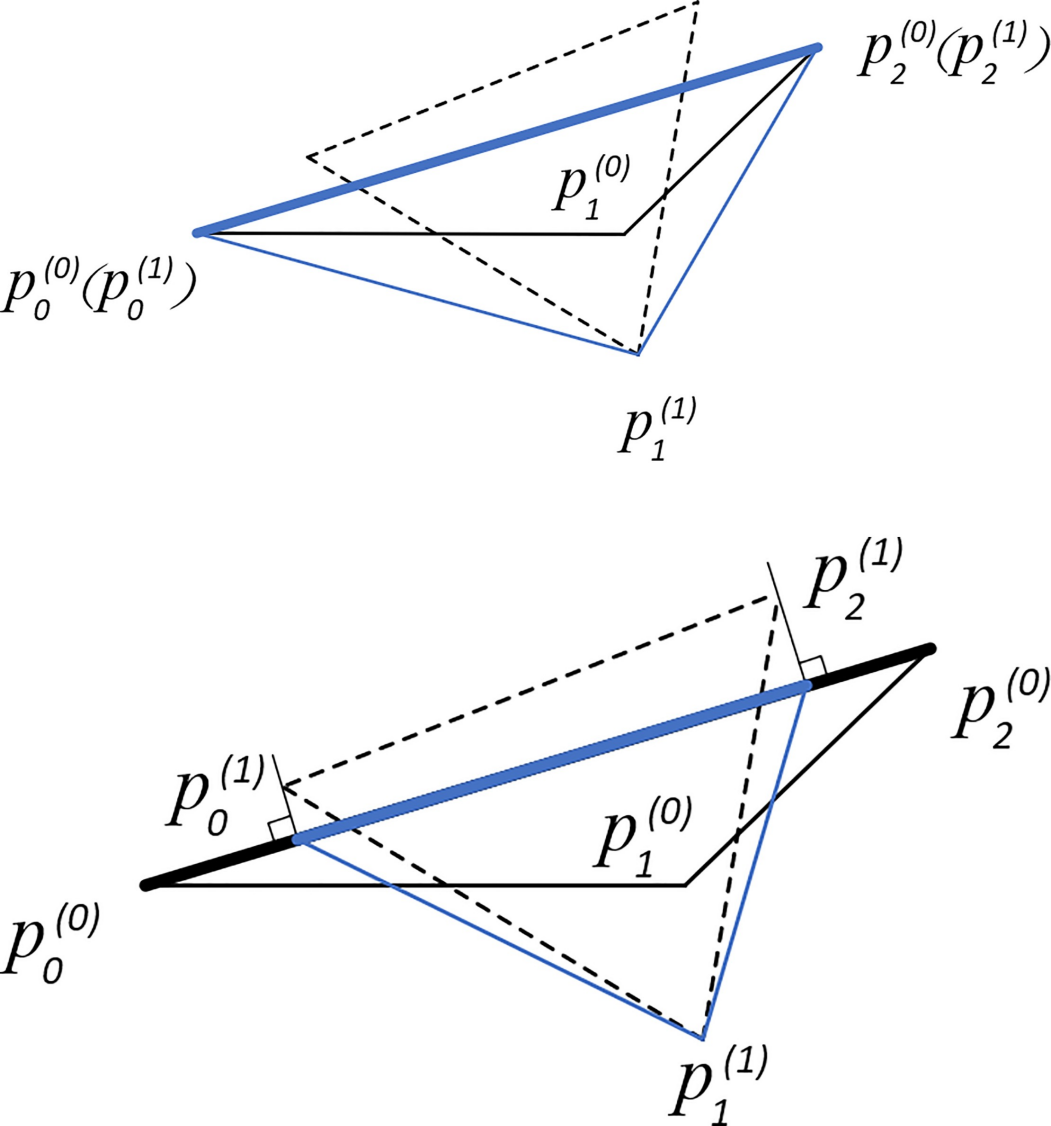
Boundary treatments of the EAS method. a) shows the way of fixing boundary nodes at their original locations; b) shows the way of moving nodes along the direction of boundary edges by projection. Original triangles are in blue solid lines; virtual triangles after smoothing without boundary treatments are in black dotted lines; actual triangles after smoothing considering boundary treatments are in blue solid lines black; and boundary edges are in heavy solid lines.

EAS method takes boundary nodes into account. Fig 5 illustrates treatments for boundary nodes, which is either fixing boundary nodes at their original positions or merely allowing them to move along the direction of boundary edges.

### 3.5 Convergence

**Fig 6:**
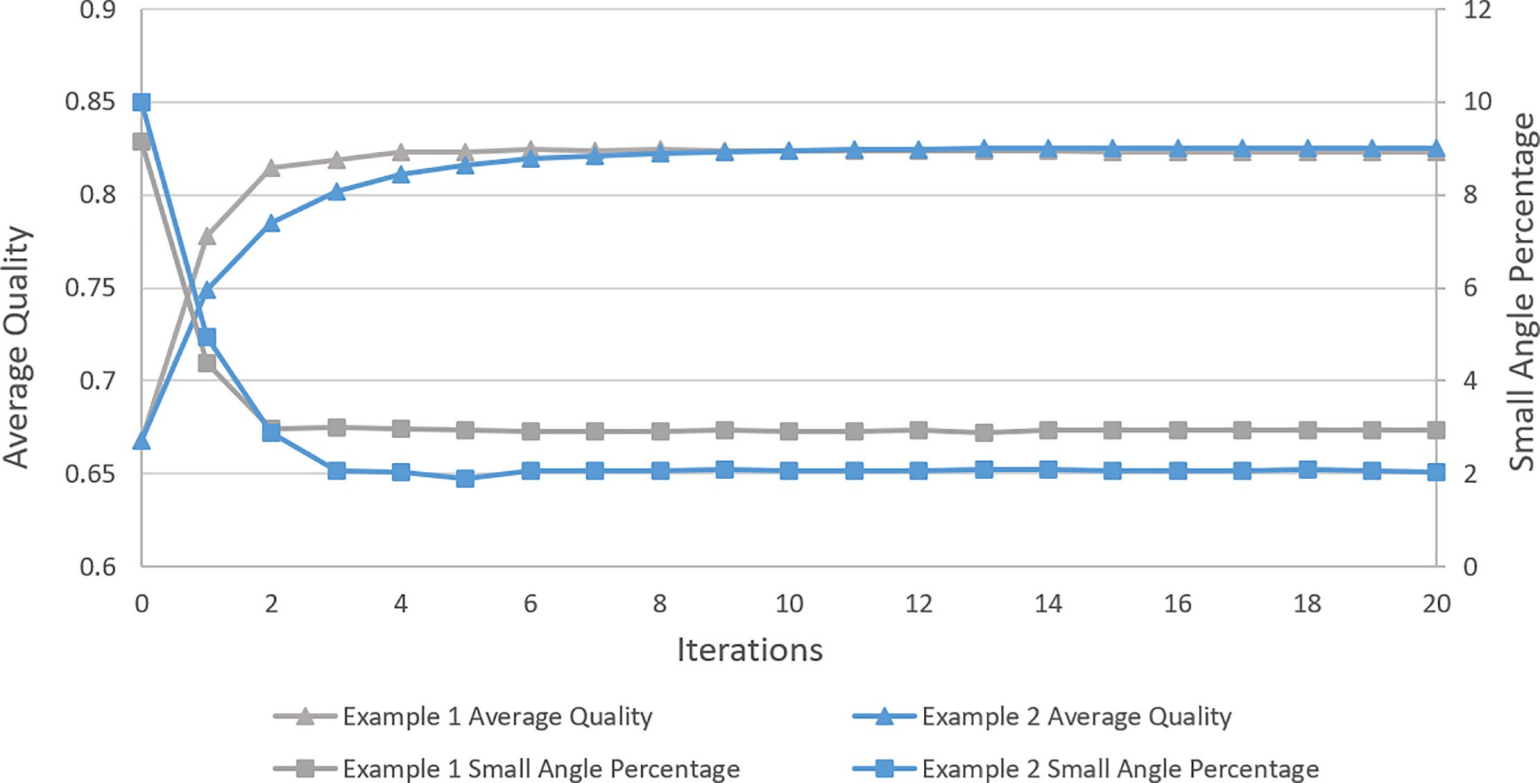
Convergence of EAS algorithm for triangular mesh.

In terms of quality versus computational cost, the number of smoothing times have to be finite. Such is an ideal situation that mesh quality is improved and stabilizes at a higher level with the less increasing smoothing time. Although it is hard to obtain a rigorous mathematical proof for the convergence of this linear system, plenty of numerical experiments are carried out to testify the convergence of EAS algorithm. Through plenty of numerical tests, EAS can reach a high performance when t has the value of 0.5. Typical demonstrations of average quality and small angle percentage (0~20°) changes are illustrated in Fig 6 where EAS method shows its performance and stability.

### 3.6 Extension to 3D surface

Given that most of the real-world models are closed surface meshes, EAS method can be extended to surface mesh smoothing. Similar to boundary edges and boundary nodes on planar meshes, there are some constrained edges and nodes called feature edges and feature nodes on closed surface meshes, respectively, which reflect some shape features such as sharp-edges, corners, grooves and so on. Netgen [27] recognizes feature edges by the means of calculating the dihedral angle of two adjacent elements sharing one common edge, which is also used in [28].

EAS method adopts the same treatments for feature nodes as adopted for boundary nodes on planar meshes which is either fixing feature nodes at their original locations or merely allowing them to move along the direction of feature edges. For higher precision, a feature node is allowed to move along the local interpolation curve constructed by its adjacent nodes.

**Fig 7:**
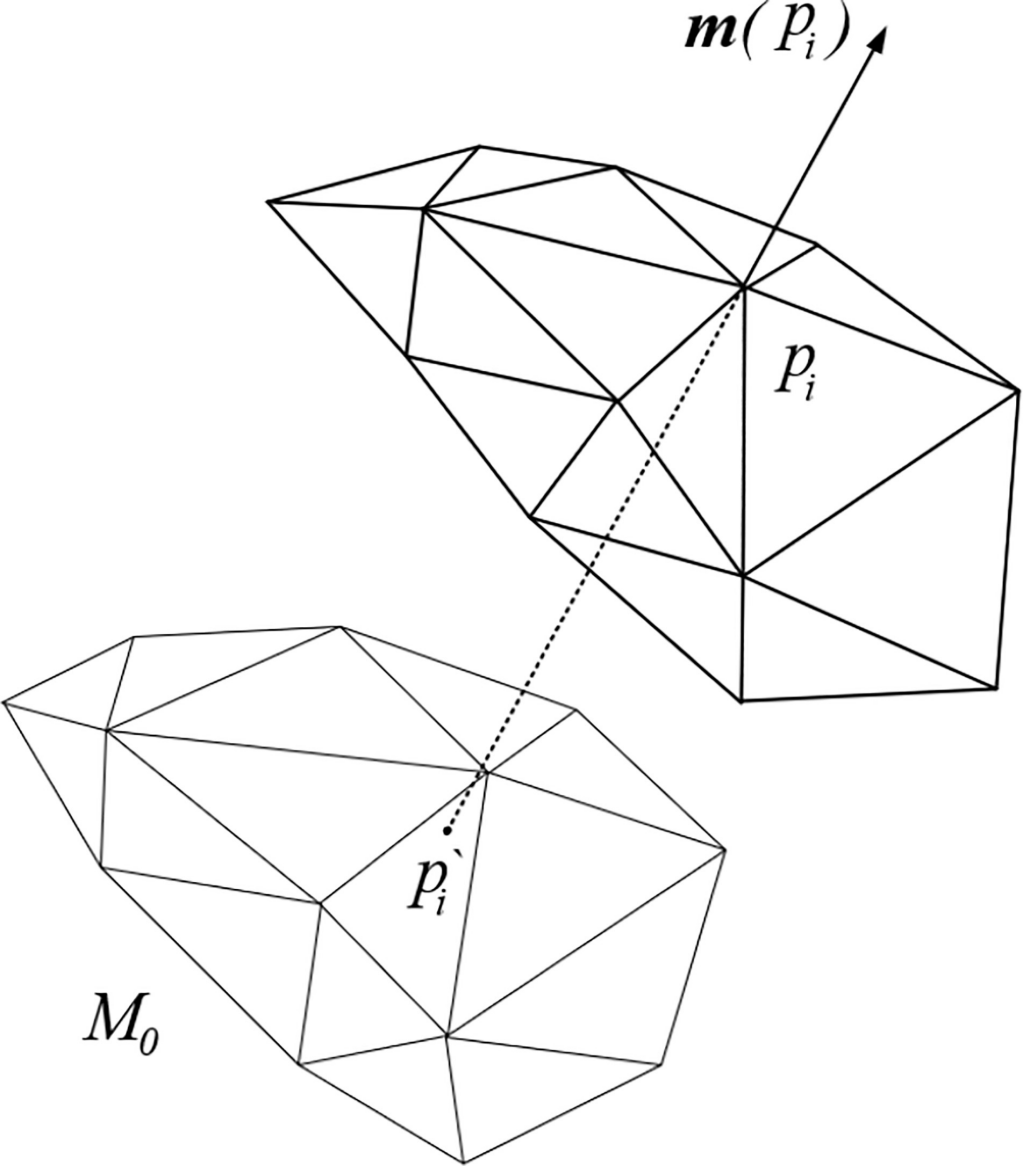
Projection operation of surface mesh smoothing.

In order to preserve original geometric information, the original surface mesh is always recorded as *M*_0_ before smoothing. Except for feature nodes, each node can obtain a mean normal based on its ring using the approach referred by [29], denoted as ***m***(*p*_*i*_) in Fig 7. After one time of smoothing, the final position of node $p_{i}^{\prime }$ is obtained through the way of projecting *p*_*i*_ derived from assembly back to the original mesh *M*_0_ along ***m***(*p*_*i*_), which is also used in Mesquite [30]. On the other hand, if high precision is required, Coon patches [28] or least-squares method can help to construct a local parametric surface piecewisely based on adjacent elements of the original mesh *M*_0_. For the nodes updated after one time of smoothing, final positions $p_{i}^{\prime }$ can be obtained by projecting *p*_*i*_ back to the local parametric surface along ***m***(*p*_*i*_) to proceed subsequent iterations.

## 4. Smoothing algorithm for polygonal mesh

### 4.1 Smoothing algorithm for quadrilateral mesh

**Fig 8:**
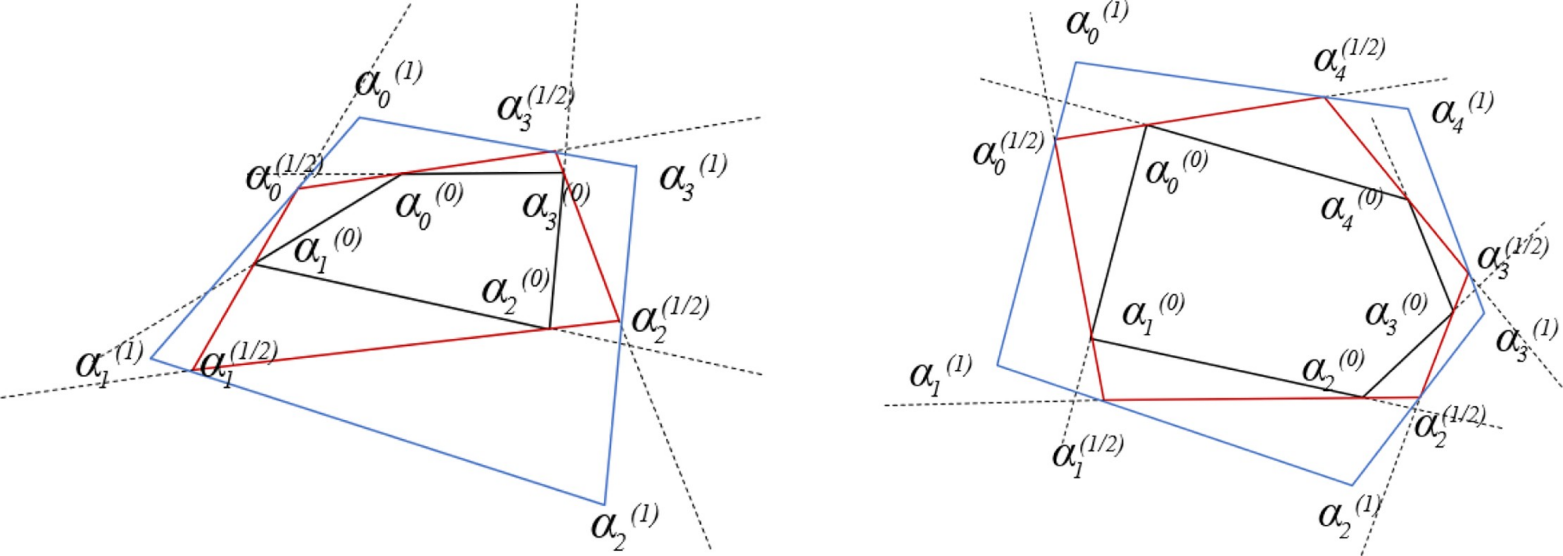
Operations of the first stage of EAS method for quadrilateral mesh. a) shows EAS transformation for quadrilateral mesh when t is 0.25; b) shows EAS transformation for pentagonal mesh when t is 0.25.

EAS smoothing algorithm can be also applied to quadrilateral mesh smoothing. As is similar to the case of triangular mesh, EAS transformation is to make each element equilateral and the final result is obtained with a weighted assembly strategy. The regularity for element transformations can be proved on the theory level and numerical experiments illustrate its convergence. The EAS smoothing for quadrilateral mesh is illustrated in Fig 8A.

$$\alpha_{m}^{\left(\frac{1}{2}\right)}=\pi -(1-t)∙{(\pi -\alpha }_{m}^{\left(0\right)})-t∙(\pi -\alpha_{mod(m+1,3)}^{\left(0\right)})$$ (9)

$$K_{+}=\left[\begin{array}{cccc} 1-t & t & 0 & 0\\ 0 & 1-t & t & 0\\ 0 & 0 & 1-t & t\\ t & 0 & 0 & 1-t \end{array}\right]$$ (10)

Similarly, interior angles at different stage are denoted as $\alpha_{m}^{\left(0\right)},\;\alpha_{m}^{\left(1/2\right)},\;\alpha_{m}^{\left(1\right)},\;m\epsilon \{0,1,2,3\}$, whose subscript is the same as the subscript of the corresponding node and the superscript stands for the current stage. Interior angles after the first substage of the first stage can be obtained as Eq 9 with the corresponding transition matrix *K*_+_ in Eq 10.

$$\left\{\alpha^{\left(1\right)}\}=K\{\alpha^{\left(0\right)}\}=\left[\begin{array}{cccc} {2t}^{2}-2t+1 & -t^{2}+t & 0 & -t^{2}+t\\ -t^{2}+t & {2t}^{2}-2t+1 & -t^{2}+t & 0\\ 0 & -t^{2}+t & {2t}^{2}-2t+1 & -t^{2}+t\\ -t^{2}+t & 0 & -t^{2}+t & {2t}^{2}-2t+1 \end{array}\right]\{\alpha^{\left(0\right)}\right\}$$ (11)

Eq 11 shows the transition matrix for the first stage. Through spectral decomposition eigenvalues are computed as 4*t*^2^−4*t*+1, 2*t*^2^−2*t*+1, 2*t*^2^−2*t*+1, 1, respectively. Like the case of triangular mesh, *λ*_3_ is the only dominant item for EAS transformation with lim_*l*→∞_*λ*_0_^*l*^ = *λ*_1_^*l*^ = *λ*_2_^*l*^ = 0. After eigenvector normalization, a series of EAS transformation can average interior angles for quadrilateral mesh from a theoretical point of view as shown in Eq 12.

$$K^{l}=\sum_{n=0}^{3}{\lambda_{n}}^{l}(\Phi I_{n}\Phi^{H})=\sum_{n=3}^{3}{\lambda_{n}}^{l}(\Phi I_{n}\Phi^{H})=\lambda_{3}∙\Phi I_{3}\Phi^{H}=\frac{1}{4}\left[\begin{array}{cccc} 1 & 1 & 1 & 1\\ 1 & 1 & 1 & 1\\ 1 & 1 & 1 & 1\\ 1 & 1 & 1 & 1 \end{array}\right]$$ (12)

**Fig 9:**
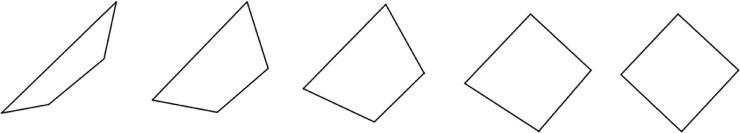
Geometric transformation process of a quadrilateral element.

**Fig 10:**
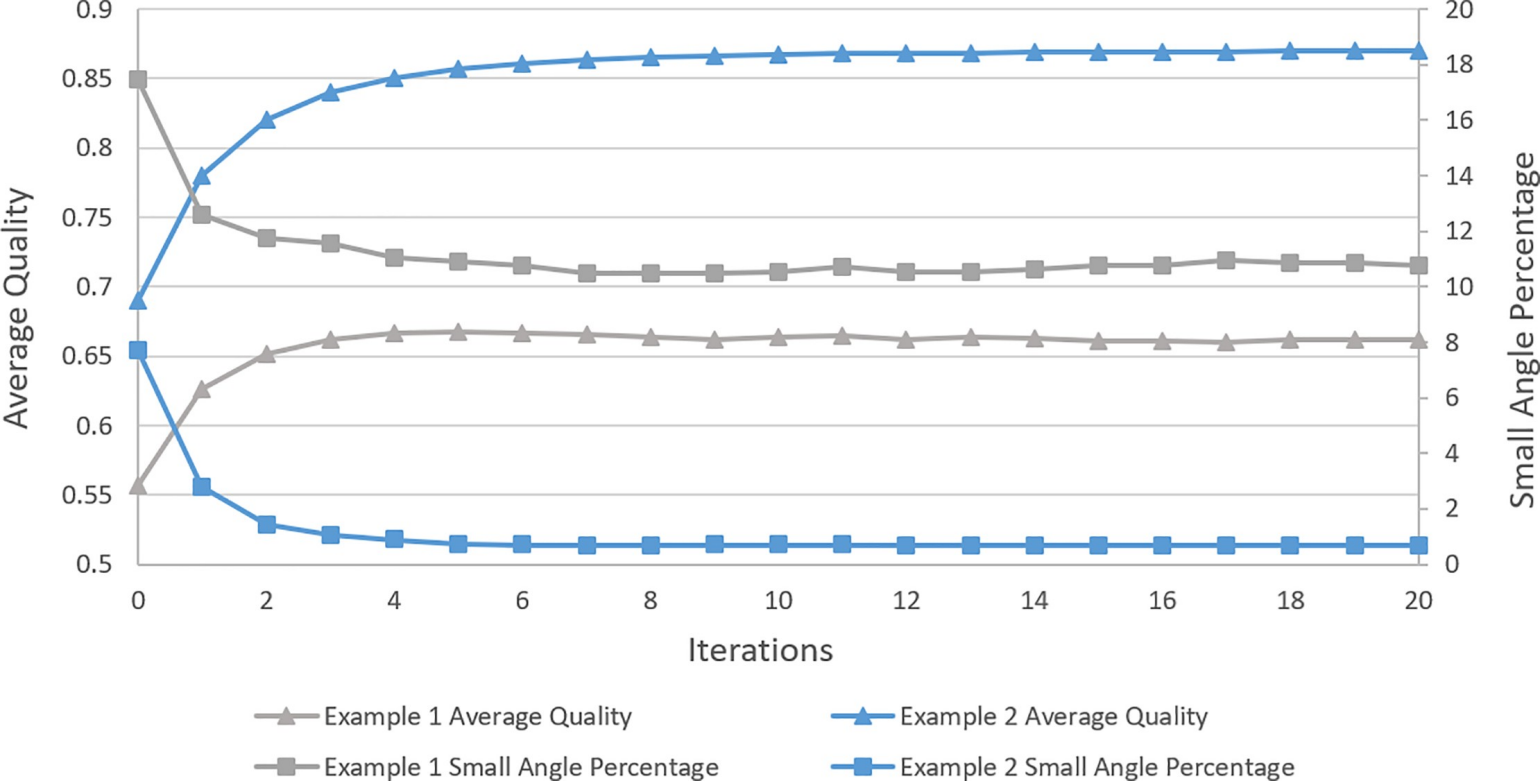
Convergence of EAS algorithm for quadrilateral mesh.

Meanwhile, the second and third stages of EAS method for quadrilateral mesh can be derived naturally and easily. As for a single quadrilateral element, Fig 9 shows the shape changing process. Numbers of experiments are conducted to testify the convergence of the proposed EAS algorithm for quadrilateral mesh. Through plenty of numerical experiments, EAS can reach a high performance for quadrilateral mesh when t takes the value of 0.5. Demonstrations of average quality and small angle percentage (0~20°) changes are shown in Fig 10.

### 4.2 Smoothing algorithm for arbitrary polygonal mesh

Considering arbitrary polygonal mesh like pentagonal mesh, EAS smoothing algorithm is also able to achieved as shown in Fig 8B. In addition, EAS method has natural applications for mixed polygonal mesh with few adjustments.

## 5. Results and discussions

This section illustrates the performance of EAS smoothing algorithm described in the previous sections. Several examples from different areas are selected for demonstration and the results are discussed. Taking generality and practicality into account, tests are only implemented on models with triangular elements and quadrilateral elements. Since EAS method is based on element geometric transformation, this section compares EAS with other existing state-of-the-art element-transformation-based methods which have influence and is widely recognized in the field of mesh smoothing. And the optimization-based method is also chosen as a comparative item because it is usually time-consuming but recognized to reach an excellent mesh quality. Smoothing methods are programmed by C++ aided by OpenMesh [31] and the optimization-based method is implemented by Mesquite. Figures are screenshot by visualizer with a STL files and VTK files.

This paper selects the ratio of two times incircle radius (2r) and circumcircle radius (R) of the triangle as quality criteria denoted as *γ* for triangular mesh. And for quadrilateral mesh, as proposed in Gambit, the preprocessor of Fluent [32], the angle between links of midpoints of opposite edges is regarded as quality criteria *γ* to measure the skewness. And there are more quality criteria in [33–35]. Quality criteria *γ* takes the value of 1 for regular element and 0 for degenerated element after normalization. Changes of node’s position may produce negative elements with inversion, which is not the concern of this paper. To avoid the problem of negative elements, each movement is accepted only if no negative element emerges, or this movement is rejected. Such constraint operation is introduced to the tests for all smoothing methods mentioned in this section.

To evaluate the global quality of a mesh model, this section selects average quality, minimum quality, minimum internal angle and small angles percentage (0~20°) as measurements. All of mentioned smoothing loops execute ten times of iterations.

**Fig 11:**
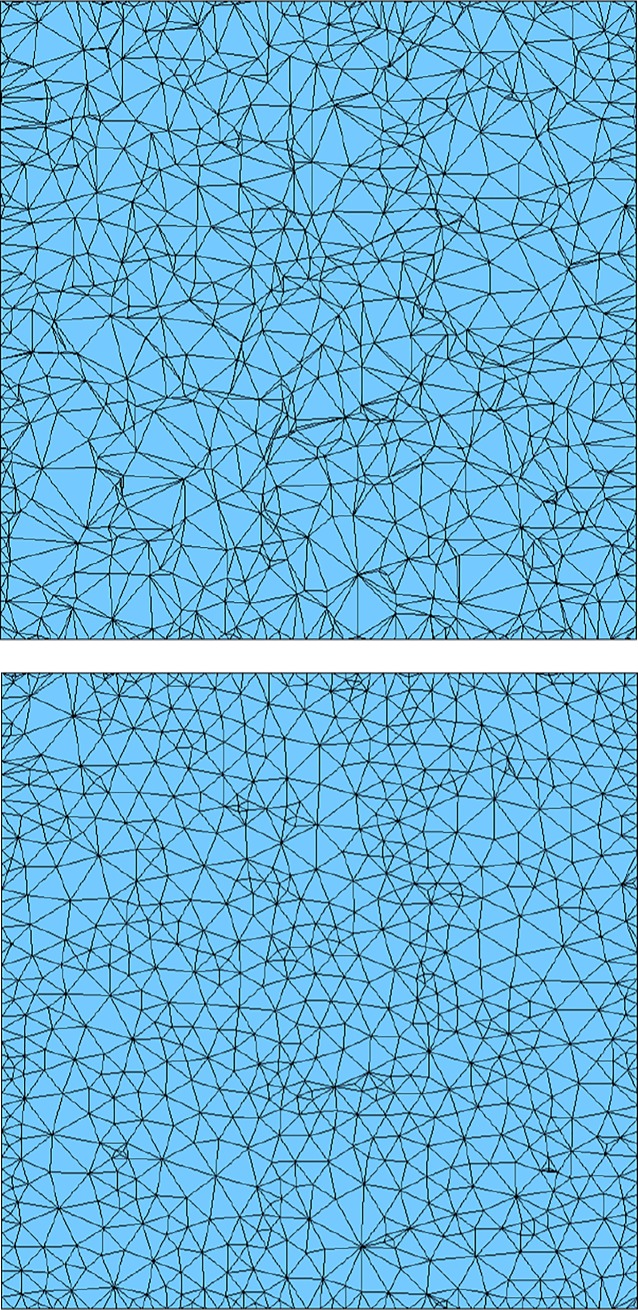
Example 1 before(a) and after(b) EAS smoothing.

**Fig 12:**
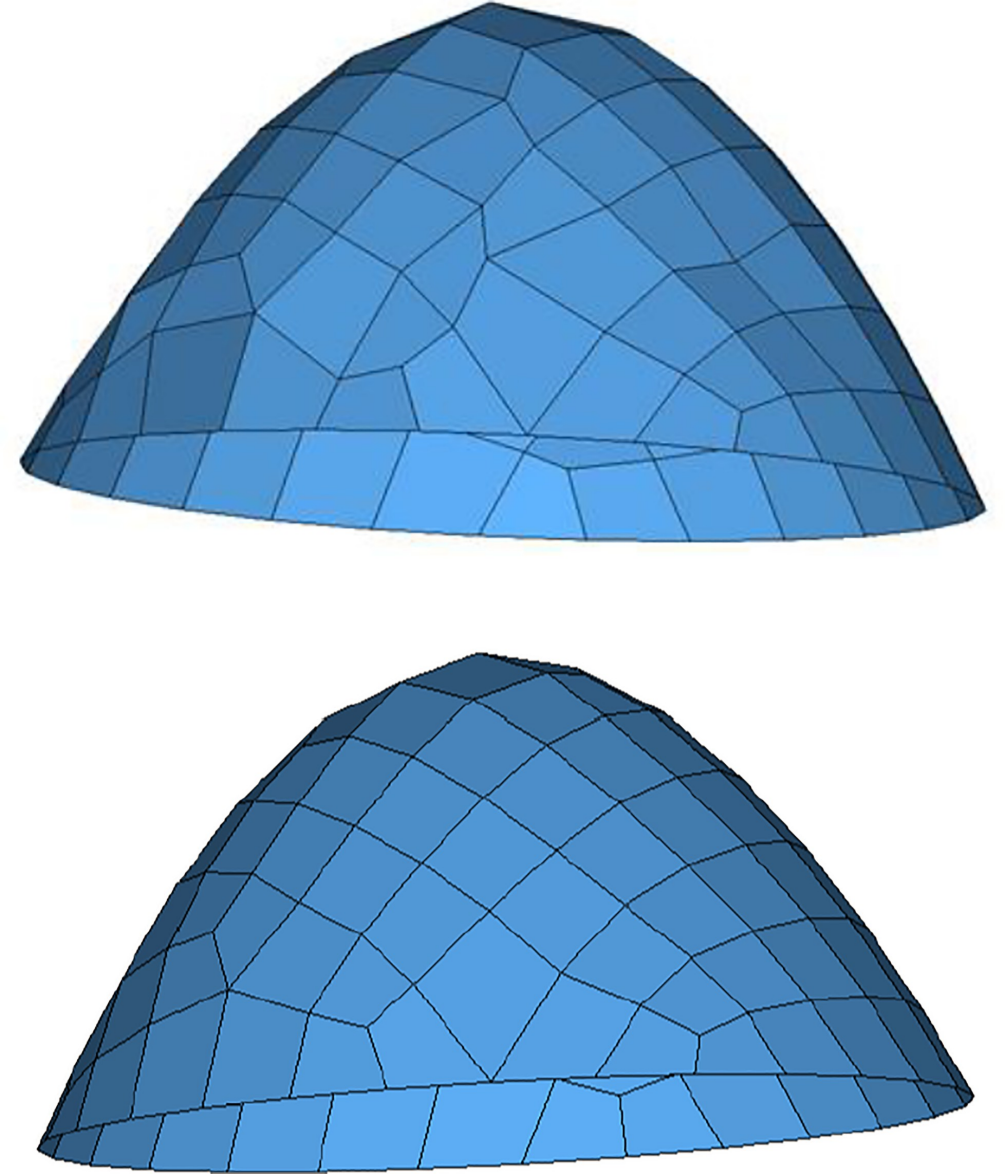
Example 2 before(a) and after(b) EAS smoothing.

The first example is a planar triangular mesh with 900 nodes and 1598 elements. Several smoothing methods are taken to improve its quality and results are listed in Table 1. It can be seen that the proposed EAS method has advantages in average quality and small angles percentage. Meshes before EAS smoothing and after EAS smoothing are demonstrated in Fig 11. The second example is a practice of quadrilateral surface mesh, whose results are listed in Table 1. Meshes before EAS smoothing and after EAS smoothing are demonstrated in Fig 12.

**Table 1:** Test results.

Mesh	Method	Average Quality	Minimum Quality	Minimum Angle(°)	Small Angles Percentage(0~20°)
Example 1	Initial Mesh	0.690	0.015	0.501	7.984
	GETme	0.868	0.023	9.524	0.836
	SSO	0.855	0.286	9.694	0.503
	EAS	0.868	0.123	5.912	0.709
	Mesquite	0.879	0.256	10.875	0.457
Example 2	Initial Mesh	0.768	0.548	4.084	0.000
	GETme	0.920	0.672	10.737	0.000
	SSO	0.913	0.738	13.026	0.000
	EAS	0.920	0.644	9.805	0.000
	Mesquite	0.934	0.742	13.364	0.000
Example 3	Initial Mesh	0.564	0.014	0.056	16.330
	GETme	0.744	0.027	1.428	1.976
	SSO	0.742	0.007	0.226	2.498
	EAS	0.745	0.027	1.039	2.081
	Mesquite	0.765	0.030	1.457	1.428
Example 4	Initial Mesh	0.628	0.083	2.944	12.874
	GETme	0.752	0.181	7.685	1.246
	SSO	0.749	0.223	10.761	0.409
	EAS	0.755	0.131	6.154	0.909
	Mesquite	0.760	0.124	10.987	0.000
Example 5	Initial Mesh	0.768	0.002	0.084	5.322
	GETme	0.846	0.114	3.476	0.736
	SSO	0.828	0.125	4.235	1.140
	EAS	0.848	0.045	2.167	0.534
	Mesquite	0.851	0.127	6.278	0.362

**Fig 13:**
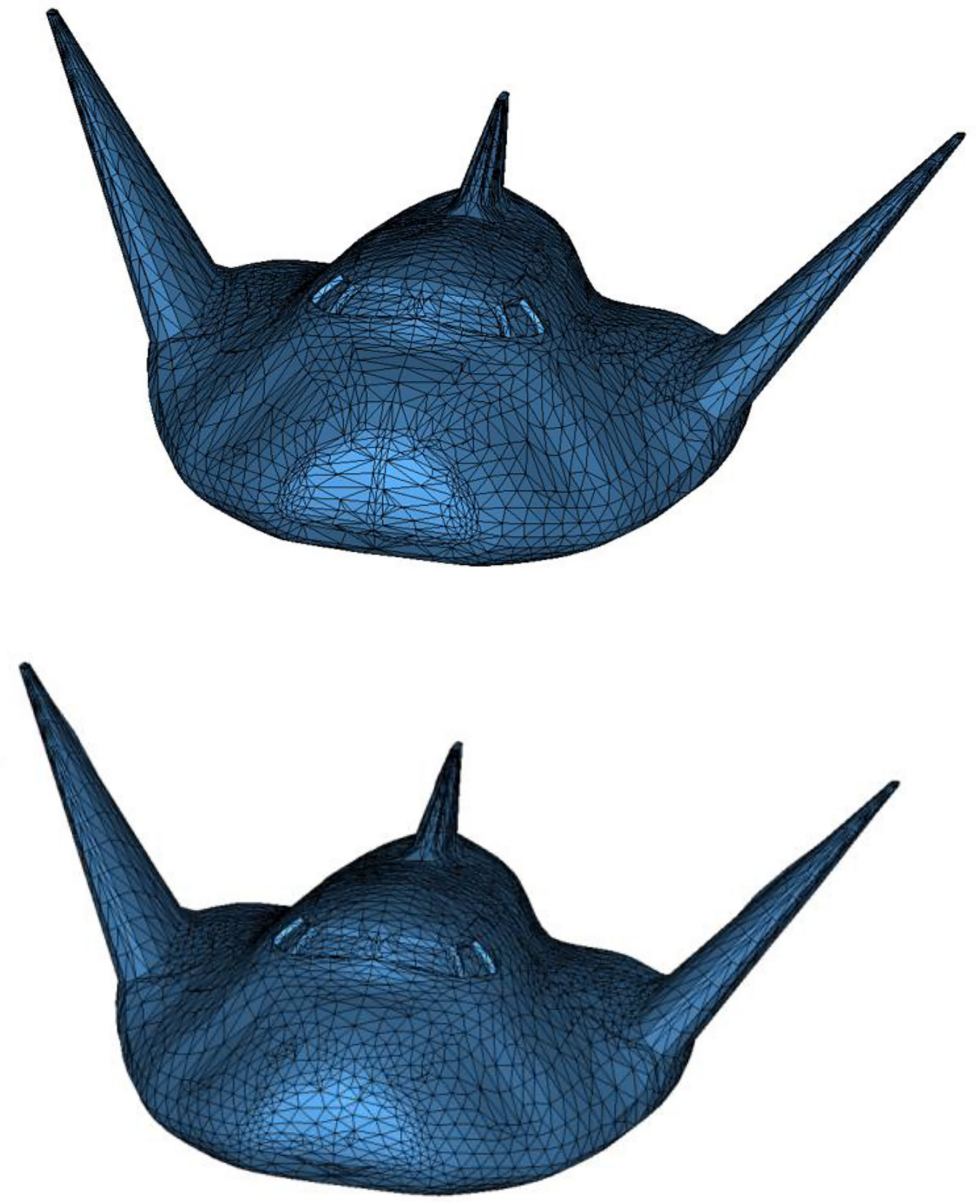
Example 3 before(a) and after(b) EAS smoothing.

Example 3 is a model of spacecraft with 4348 nodes and 8413 triangular elements. The initial quality is poor and results are presented in Table 1. Meshes before EAS smoothing and after EAS smoothing are shown in Fig 13. Clearly, as for element transformation-based methods, EAS method still has advantages in average quality compared with other methods and GETme has better performance in minimum angle and angle distribution.

**Fig 14:**
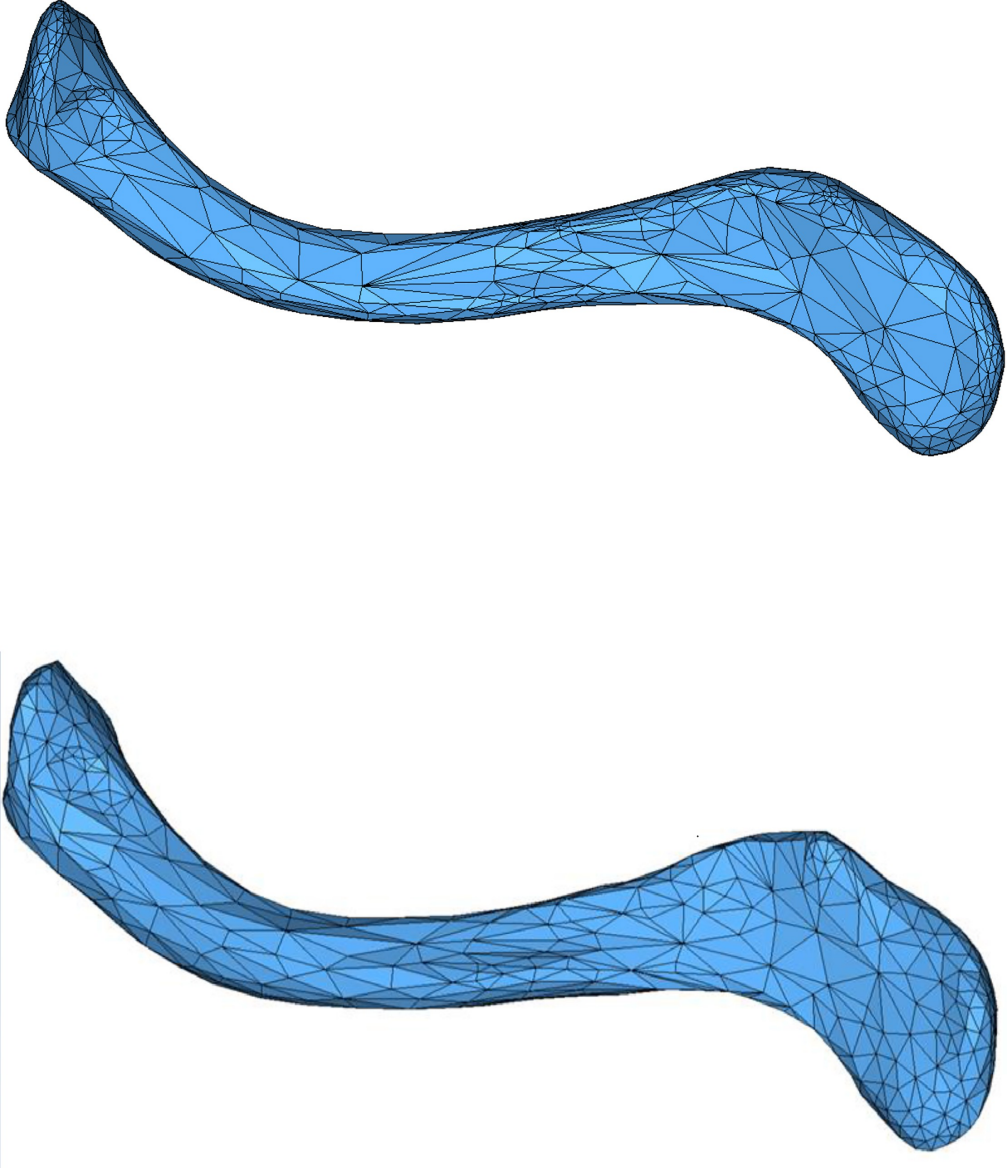
Example 4 before(a) and after(b) EAS smoothing.

The fourth example is a triangular mesh of shoulder blade with 589 nodes and 1174 elements, as shown in Fig 14A. The smoothed mesh is presented in Fig 14B. As listed in Table 1, smoothing operations have improved mesh quality remarkably in different measurements. Among element-transformation-based methods, EAS method can obtain better average quality while SSO has better angle distribution.

**Fig 15:**
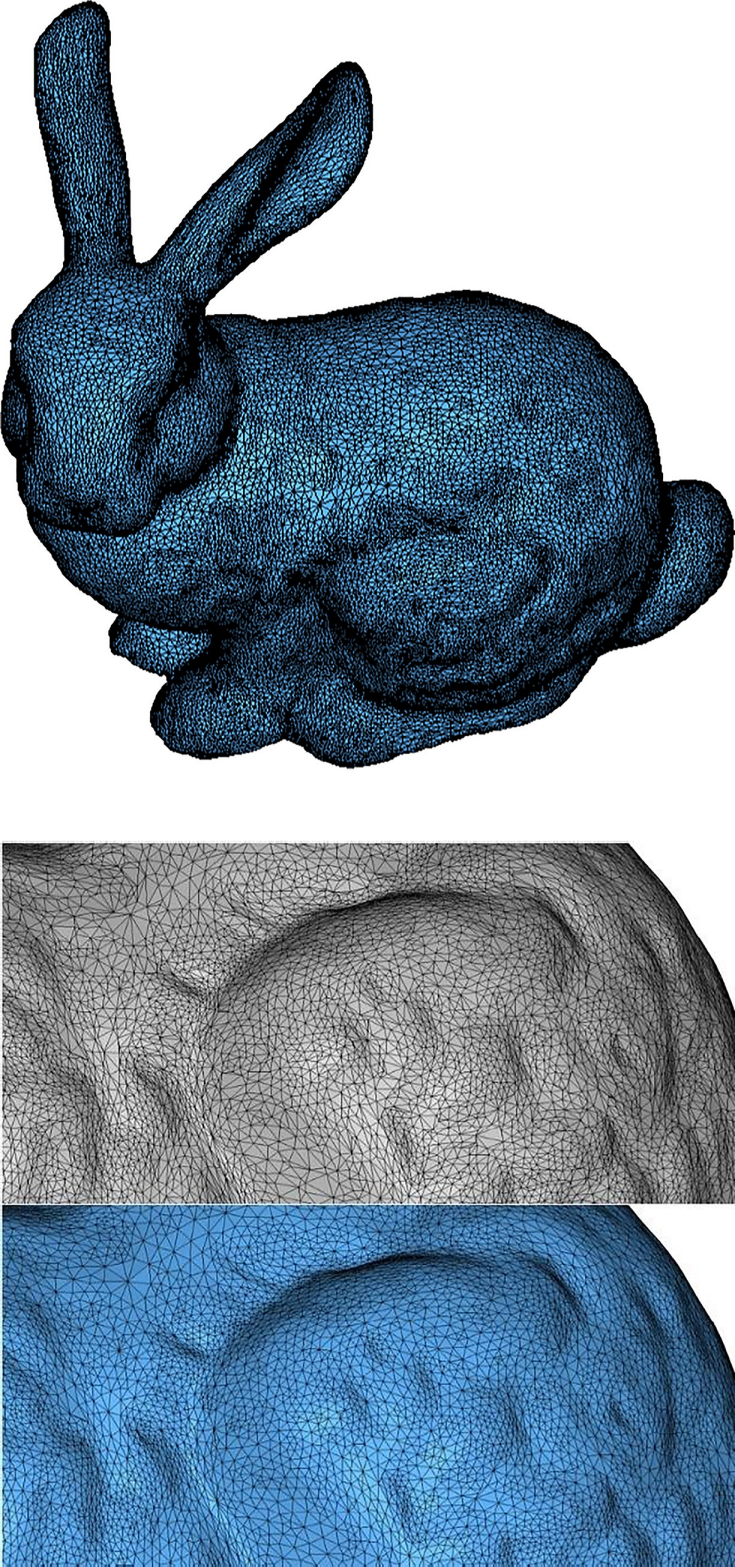
a) Initial mesh of Example 5; b) local comparison of mesh before(up) and after(down) EAS smoothing.

**Fig 16:**
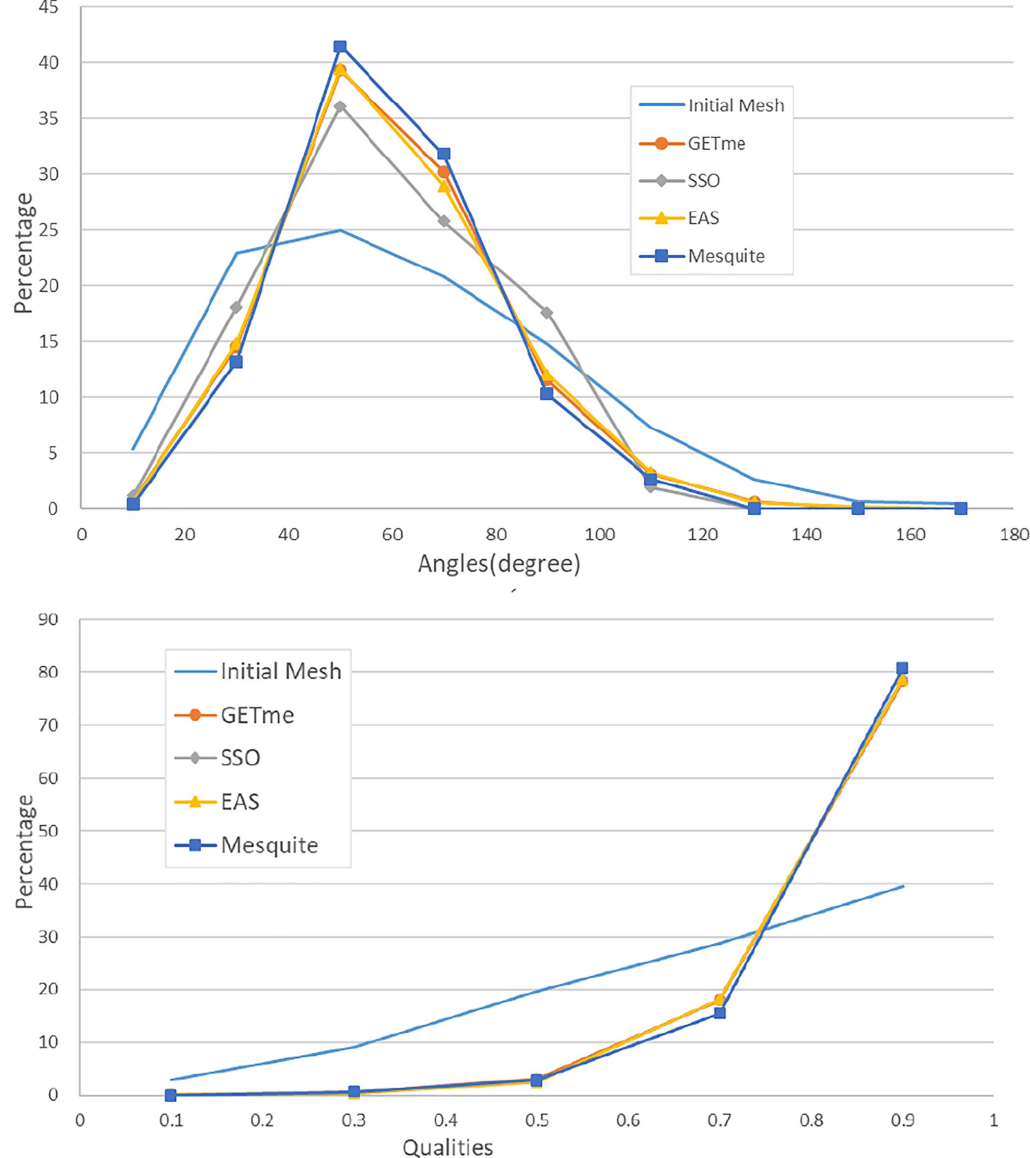
a) Distribution of qualities in Example 5; b) Distribution of angles element in Example 5.

The fifth example is a Stanford bunny of triangular mesh containing 43199 nodes and 86394 elements. EAS method improves the average quality and reduces small angles percentage significantly as presented in Table 1 and Fig 15. Through zooming in, it can be seen that EAS exerts positive effects for mesh quality improvement noticeably in Fig 15. And Fig 16 illustrates the distribution of element interior angles and element qualities.

**Fig 17:**
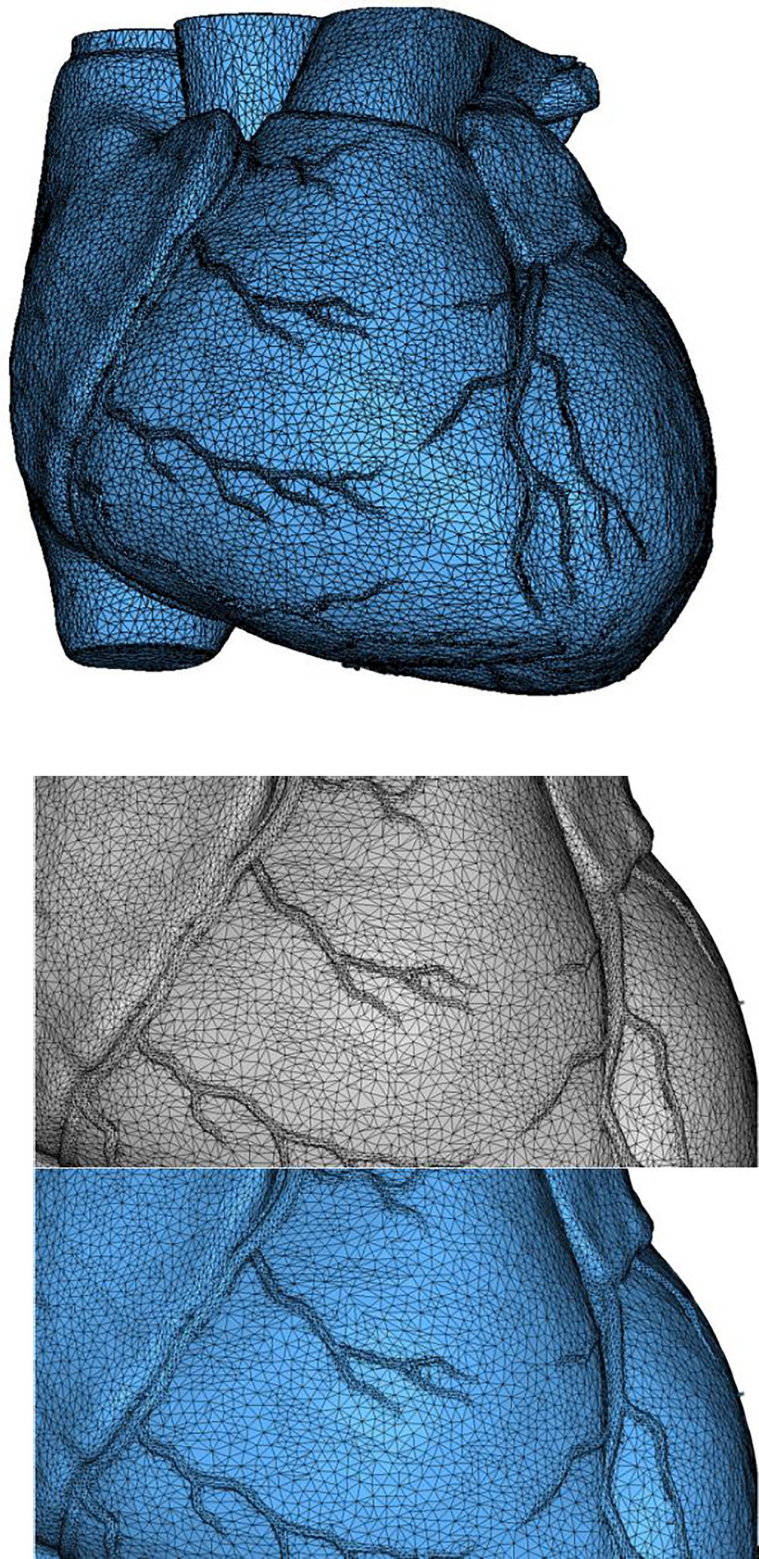
a) Initial mesh of Example 6; b) local comparison of mesh before(up) and after(down) EAS smoothing.

**Fig 18:**
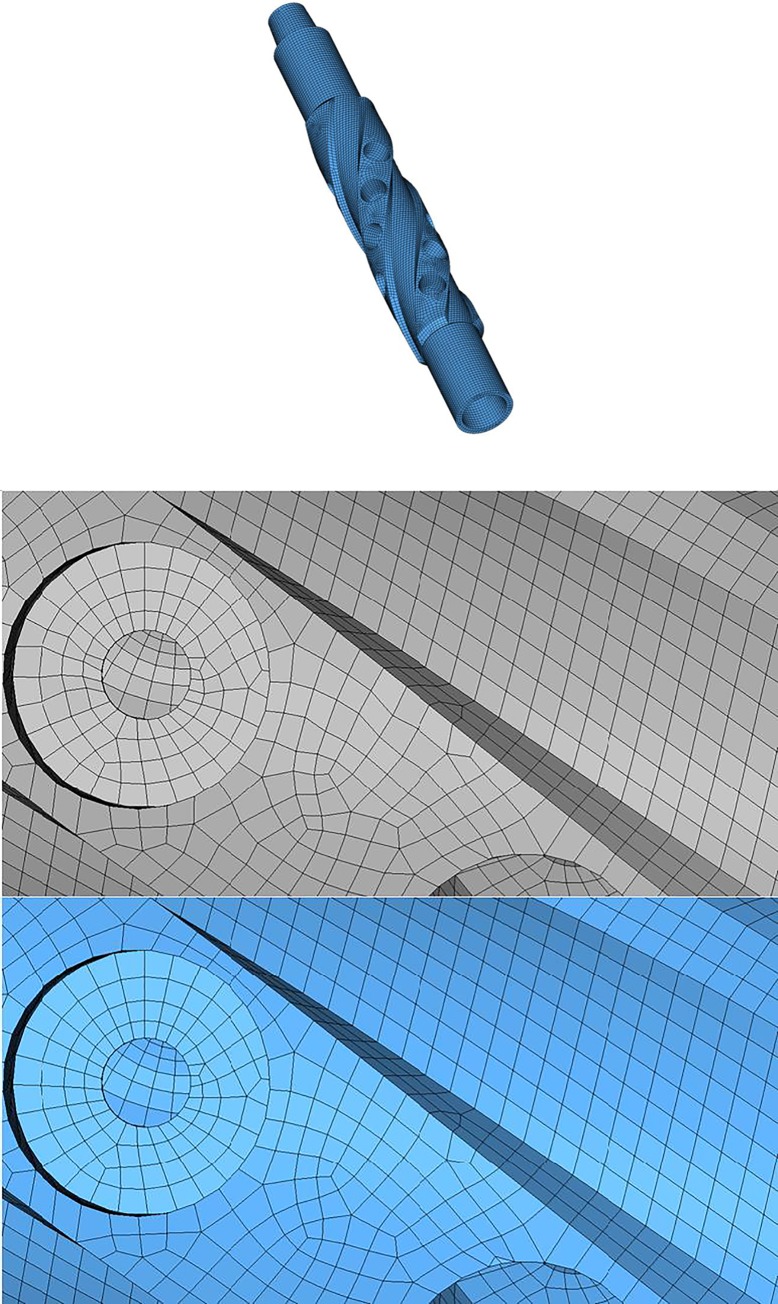
a) Initial mesh of Example 7; b) local comparison of mesh before(up) and after(down) EAS smoothing.

Example 6 is a scanned heart model of triangular mesh with 34505 nodes and 70000 elements. The smoothed mesh and a part of the mesh before and after smoothing are illustrated in Fig 17, and the results are listed in Table 2. The last example is a spline of quadrilateral-dominant mesh as shown in Fig 18, composed of 26767 nodes and 26830 elements. Results before and after EAS smoothing are listed in Table 2.

**Table 2:** Test results of complex models.

Mesh	Method	Average Quality	Minimum Quality	Minimum Angle(°)	Small Angles Percentage(0~20°)
Example 6	Initial Mesh	0.868	0.016	0.525	9.483
	EAS	0.924	0.023	0.773	1.723
Example 7	Initial Mesh	0.822	0.620	41.187	0.000
	EAS	0.916	0.767	50.817	0.000

## 6. Conclusions and future works

This paper presents an algorithm for 2D planar and surface mesh smoothing using element geometric transformation. This algorithm conducts geometric transformation independently for each element based on an exterior-angle-split process, and then assemble them into an integral mesh of high quality with a weighted strategy.

The presented method has satisfactory outcomes. From a theoretical point of view, it can be proved that EAS method can make an element equilateral. And plenty of experiments are carried out to testify the convergence of EAS algorithm from a global perspective. With simple and appropriate adjustments, this method is capable of being extended to surface meshes of arbitrary polygonal element. And numerical experiments show that EAS method has effectiveness in mesh smoothing evidently. Conclusively, the proposed EAS smoothing algorithm has very simple transformation rules and leads to significant improvement for mesh quality.

EAS method is a novel geometric smoothing method, and combination with topological methods is where one of the potentials resides in the future. On the other hand, EAS method introduced in this paper remains at the level of polygonal mesh. Whereas, polyhedral mesh also has vital applications especially in industrial simulation, which puts forwards new goals and requirements for this method. Further research will naturally focus on its combination with topological methods and extension to polyhedral mesh.

## References

[1] Newman T.S. and Yi H., A survey of the marching cubes algorithm. Computers & Graphics, 2006 30(5): p. 854–879.

[2] Si H., TetGen, a Delaunay-Based Quality Tetrahedral Mesh Generator. ACM Transactions on Mathematical Software, 2015 41(2): p. 1–36.

[3] Löhner R. and Parikh P., Generation of three-dimensional unstructured grids by the advancing-front method. International Journal for Numerical Methods in Fluids, 1988 8(10): p. 1135–1149.

[4] Lo S.H., A new mesh generation scheme for arbitrary planar domains. International Journal for Numerical Methods in Engineering, 1985 21(8): p. 1403–1426.

[5] Shang F G.Y., Guo Y, Hexahedral mesh generation via constrained quadrilateralization. PLOS ONE, 2017 12(5): p. e0177603 10.1371/journal.pone.0177603 28542355 PMC5436764

[6] Freitag Diachin L., et al, A comparison of two optimization methods for mesh quality improvement. Engineering with Computers, 2006 22(2): p. 61–74.

[7] Chen Z., Tristano J.R., and Kwok W., Construction of an objective function for optimization-based smoothing. Engineering with Computers, 2004 20(3): p. 184–192.

[8] Freitag L.A. and Ollivier-Gooch C., A Cost/Benefit Analysis of Simplicial Mesh Improvement Techniques as Measured by Solution Efficiency. International Journal of Computational Geometry & Applications, 2012 10(04): p. 361–382.

[9] Plassmann L.A.F.P., Local Optimization-Based Simplicial Mesh Untangling And Improvement. International Journal for Numerical Methods in Engineering, 2000.

[10] Field D.A., Laplacian smoothing and Delaunay triangulations. Communications in Applied Numerical Methods, 1988 4(6): p. 709–712.

[11] HANSBO P., Generalized Laplacian smoothing of unstructured grids. COMMUNICATIONS IN NUMERICAL METHODS JN ENGINEERING, 1995 11: p. 455–464.

[12] Vollmer J., Mencl R., and Muller H., Improved Laplacian Smoothing of Noisy Surface Meshes. Computer Graphics Forum, 1999 18(3): p. 131–138.

[13] Mao Zhihong, M.L., Zhao Mingxi, and Li Zhong, A Modified Laplacian Smoothing Approach with Mesh Saliency. Smart Graphics, 6th International Symposium, SG 2006, Vancouver, Canada, July 23–25, 2006, Proceeding, 2006.

[14] Liu T., et al, Quality improvement of surface triangular mesh using a modified Laplacian smoothing approach avoiding intersection. PLoS One, 2017 12(9): p. e0184206 10.1371/journal.pone.0184206 28886110 PMC5590917

[15] Shimada T.Z.a.K., An Angle-Based Approach to Two-Dimensional Mesh Smoothing. New Orleans, 2000.

[16] Vartziotis D., et al, Mesh smoothing using the Geometric Element Transformation Method. Computer Methods in Applied Mechanics and Engineering, 2008 197(45–48): p. 3760–3767.

[17] Vartziotis D. and Wipper J., The geometric element transformation method for mixed mesh smoothing. Engineering with Computers, 2009 25(3): p. 287–301.

[18] Sun S., Zhang M., and Gou Z., Smoothing Algorithm for Planar and Surface Mesh Based on Element Geometric Deformation. Mathematical Problems in Engineering, 2015 2015: p. 1–9.

[19] Wang D., et al, Enhanced remeshing from STL files with applications to surface grid generation. Communications in Numerical Methods in Engineering, 2006 23(3): p. 227–239.

[20] Frey P.J. and Borouchaki H., Geometric surface mesh optimization. Computing and Visualization in Science, 1998 1(3): p. 113–121.

[21] Sun J.L.a.S., Small Polyhedron Reconnection A New Way to Eliminate Poorly-Shaped Tetrahedra. 2006.

[22] Acharya B.P. and Acharya M., Mesh Optimization Based on the Centroid Voronoi Tessellation International Journal of Computer Mathematics, 2005 82(1): p. 125–129.

[23] Huang Y., Qin H., and Wang D., Centroidal Voronoi tessellation-based finite element superconvergence. International Journal for Numerical Methods in Engineering, 2008 76(12): p. 1819–1839.

[24] Lévy B. and Liu Y., LpCentroidal Voronoi Tessellation and its applications. ACM Transactions on Graphics, 2010 29(4).

[25] L.Chen, Mesh smoothing schemes based on optimal Delaunay triangulations. Proceedings of the 13th International Meshing Roundtable, pp. 109–120, Sandia National Laboratories, 2004., 2004.

[26] Chen L. and Holst M., Efficient mesh optimization schemes based on Optimal Delaunay Triangulations. Computer Methods in Applied Mechanics and Engineering, 2011 200(9–12): p. 967–984.

[27] Schoberl, J., Netgen. https://ngsolve.org/.

[28] Béchet E., Cuilliere J.C., and Trochu F., Generation of a finite element MESH from stereolithography (STL) files. Computer-Aided Design, 2002 34(1): p. 1–17.

[29] Belyaev H.Y.Y.O.A., Mesh Smoothing via Mean and Median Filtering Applied to Face Normals. IEEE Proceedings of the Geometric Modeling and Processing, 2002.

[30] Freitag L.L., T, Knupp P, MESQUITE design: issues in the development of a mesh quality improvement toolkit. 2002.

[31] University, R.A., OpenMesh. http://www.openmesh.org/.

[32] ANSYS Fluent. https://www.ansys.com/products/fluids/ansys-fluent.

[33] Daniel S.H, L., Finite Element Mesh Generation, CRC Press, 2015.

[34] Knupp and P.M.J.S.J.o.S. Computing, Algebraic Mesh Quality Metrics. SIAM Journal on Scientific Computing. 23(1): p. 193–218.

[35] Shewchuk J.R., What-is-a-good-linear-element-interpolation—conditioning—and-quality-measures. 2002.

